# Molecular Detection and Characterization of Bovine Diarrhea Virus (BVDV) in Aborted Fetuses and Semen Samples from Paraguay

**DOI:** 10.3390/v17101295

**Published:** 2025-09-24

**Authors:** María Fátima Rodriguez Valinotti, Eva Megumi Nara Pereira, Magaly Martinez Pereira, Rosmary Rodriguez Valinotti, Antonio Rodriguez Sanchez

**Affiliations:** 1 CEDIVEP, Veterinary Diagnostic Center of Paraguay, San Lorenzo 111422, Paraguay; r.rodriguez@cedivep.com.py (R.R.V.); arodriguez@cedivep.com.py (A.R.S.); 2 Molecular Biology and Biotechnology Department, Instituto de Investigaciones en Ciencias de la Salud, Universidad Nacional de Asunción, San Lorenzo 111422, Paraguay; enara@iics.una.py (E.M.N.P.); magaly.martinez@gmail.com (M.M.P.)

**Keywords:** BVDV, *Pestivirus* RT-PCR, Paraguay

## Abstract

Bovine viral diarrhea virus (BVDV) is the most prevalent pathogen in cattle and causes significant economic losses due to its severe clinical manifestations. It belongs to the family Flaviviridae and is distributed in species A, B, and H within the genus *Pestivirus*. The objective of this study was to detect and characterize BVDV using molecular techniques (RT-PCR) in semen and aborted fetuses samples that were sent to the CEDIVEP (Veterinary Diagnostic Center of Paraguay) laboratory. Seventy-three samples of semen from bulls were analyzed, and 54.7% of the samples were positive for *Pestivirus A*. The presence of *Pestivirus A* and *H* was detected in 2/8 spontaneously aborted fetuses. The genotypes of four individual samples of type *A* and four samples of type *H* organs were confirmed by partial sequencing of the 5-UTR region. The presence of BVDV was confirmed by molecular techniques for the first time in our country through its detection in different types of samples, as well as the presence of two genotypes. This suggests that the circulation of this virus can cause significant losses in cattle production in Paraguay.

## 1. Introduction

Bovine viral diarrhea virus (BVDV) is a diverse group of viruses responsible for causing disease in ruminants and belongs to the Pestivirus family Flaviviridae. Is a prevalent pathogen in cattle worldwide, causing significant economic losses due to a variety of clinical manifestations and is the subject of several mitigation and eradication schemes globally [1,2].

Bovine *Pestiviruses* comprise three well-known viral species; *Pestivirus A* (formerly Bovine viral diarrhea virus type 1, BVDV-1) *Pestivirus B* (formerly Bovine viral diarrhea virus type 2, BVDV-2) and *Pestivirus H* (formerly Bovine viral diarrhea virus type 3, BVDV-3—HobiPev) [3]. BVDV is considered, not as a single entity, but as a heterogeneous group of related viruses that differ in their antigenicity, cytopathogenicity and virulence [4]. The comparison of the highly conserved regions, such as the 5′UTR, with the highly variable regions, including Npro and E2, has facilitated the classification and phylogenetic analysis of *Pestiviruses* into distinct genotypes and subgenotypes [3,5,6]. Consequently, 21 subgenotypes have been identified for *Pestivirus A* (1a–1u), whereas 4 subgenotypes have been recognized for *Pestivirus B* (2a–2d). Based solely on published sequences, the number of isolates described for *Pestivirus A* is significantly greater than those for *Pestivirus B* (88.2% versus 11.8%). However, the relative proportions of each virus vary considerably depending on the country or region of origin [5]. Genetic and antigenic characterization suggest that *Pestivirus H* is the most divergent pestivirus identified in cattle to date, and phylogenetic analysis of *Pestivirus H* has resulted in at least five subgroups (a–e) [7].

Infection with BVDV has a major economic impact and leads to substantial costs to the private-public sector through decreased reproductive performance as direct losses and increased control efforts as indirect losses [8], which vary within and between countries. Direct losses due to BVDV infection are related to increased morbidity and mortality due to immunosuppression, reduced first-service conception, early embryonic death, congenital deformities, extended calving intervals, and reduced milk yield. The average direct losses per naïve dairy cow were higher than those per beef cows [9].

BVDV is endemic worldwide, with different genotypes prevailing according to different geographical regions [5,10].

The presence and molecular identification of viral strains circulating in South America have been reported in Peru, Chile, and Argentina, where *Pestivirus A* is the most prevalent genotype, specifically subgenotype 1b [5]. However, in Uruguay, Colombia, and Brazil, the most prevalent strain was subgenotype 1a [5,11,12]. *Pestivirus A* is considerably more frequent in published sequences than *Pestivirus B* [5]. Strains of *Pestivirus H* were identified in Brazil and Argentina [13,14,15].

Livestock activity is one of the most important economic activities in Paraguay, and the cattle herd is estimated at 13 million [16]. Animal health is one of the factors that have a direct impact on this activity, which is affected by the presence of BVDV in any type of livestock farm. Although there are antecedents on the presence of this virus in our country [17] to obtain the first molecular data of this virus in Paraguay, the aim of this study was to identify and genotype the BVDV in fetuses and semen from different establishments in Paraguay.

## 2. Materials and Methods

### 2.1. Samples and Study Population

Samples from eight fetuses of different gestational ages [from the first to the third trimester of gestation) from clinical cases of spontaneous abortion in establishments in the departments of Boquerón, Caaguazú, Itapúa, Misiones, and Pte. Hayes were analyzed. Samples of heart (7/8), lung (6/8), liver (7/8), brain (telencephalon) (6/8), auricular cartilage (8/8), abdominal cavity fluid (4/8), and pericardial fluid (6/8) were collected through necropsy for molecular detection of BVDV and its genotyping. Samples were not taken from all tissues in all fetuses because some samples were in process of autolysis, and in other cases, only some organs were received from aborted fetuses sent to the laboratory. The tissue samples were sectioned into 2 cm × 2 cm ieces, stored, identified in Eppendorf tubes, and frozen at −80 °C until processing.

Seventy-three samples of fresh bovine semen from bulls used for reproductive purposes (natural mating or artificial insemination) were obtained by electroejaculation. Data on breed, age, and precedence were recorded, neither clinical signs were observed, nor was the vaccination status recorded. The samples were collected from the departments of Alto Paraguay, Boquerón, Caaguazú, and Cordillera from eight livestock establishments of animals intended for bovine reproduction. Five milliliters of semen and 5 mL of serum from the same animal were sent to the laboratory. Serum samples from the bulls were tested for the detection of antibodies by the ELISA method (Civtest bovis BVD/BD P80, Hipra, Amer, Spain) and seminal samples were performed to RT-PCR.

To determine the agreement between both techniques, the Kappa index was used using the Epidat Version 4.2 program. To assess the result, it was compared with the Kappa index: Kappa = 1 (Perfect agreement), Kappa > 0.8 (Very good agreement), Kappa > 0.6 (Good agreement), Kappa > 0.4 (Acceptable agreement), Kappa > 0.2 (Weak agreement). Kappa < 0.2 (Poor Concordance).

### 2.2. RNA Extraction and RT-PCR

Viral RNA from organ samples was extracted through the commercial miRcute miRNA isolation kit (Tiangen, Beijing, China). The analysis was performed following the manufacturer’s instructions. The cDNA was prepared from the extracted RNA, so that it serves as a template for the PCR of different viral target genes, 25 µL volume reaction; 50 ng/µL of random primers, 10 mM DNTP (Takara, Kusatsu, Japan), 5 µL GoScript 5X Reaction Buffer (Promega, San Luis Obispo, CA, USA), 0.3 µL GoScript Reverse transcriptase (Promega, San Luis Obispo, CA, USA), UltraPure DNase/RNase-Free Distilled Water (Invitrogen, Carlsbad, CA, USA) and 5 µL of RNA. The cycling conditions were as follows: 25 °C for 10 min, 42 °C for 60 min, 85 °C for 5 min. For RT-PCR, the 5′UTR region was amplified with specific primers HCV90-3689 F and R for *Pestivirus A*, BVDV-2#3-F and R for *Pestivirus B*, and N2-R5-F and R for *Pestivirus H*, according to Monteiro et al. [18]. For the extraction of RNA from seminal and serum samples, we used the method described by Boom et al. [19] with modifications made prior to the process according to Alarcon-Zuñiga et al. [20]. cDNA and RT-PCR were performed as described for the organ samples. For the purpose of conducting cDNA and RT-PCR analyses, the positive control strain LREF-040/BVD Singer, genotype 1, was courteously provided by the Department of Molecular Biology at SENACSA (National Service for Animal Quality and Health). Furthermore, cDNA from three distinct viral genotypes (*A*, *B*, and *H*) served as positive controls for RT-PCR, kindly donated by Dr. Eduardo Flores Furtado and Dr. Walter Cardozo from the Faculty of Veterinary Sciences at the Federal University of Santa Maria, Rio Grande do Sul, Brazil.

### 2.3. Sequencing

Eight samples were selected, representing each type of sample analyzed (semen and fetal organs) and the different genotypes. Intense bands observed in the agarose gels and when quantified had a value greater than 10 ng/μL were selected. The following samples were chosen: four samples corresponding to *Pestivirus A* (two semen and two fetuses) and four positive samples for *Pestivirus H* (4 organs of fetuses). The amplicons obtained were quantified using the Qubit quantifier (Thermo Fisher/Invitrogen, Carlsbad, CA, USA) using the highly sensitive quantification reagent. Products with the correct band size and required concentration were purified and sequenced using Sanger technique (Macrogen, Seoul, Republic of Korea).

### 2.4. Bioinformatic Analysis

The chromatograms obtained were cleaned by removing the illegible regions at the end. The sequences were analyzed using the Basic Local Alignment Tool (BLAST) [21] to confirm that they were the target genes. A phylogenetic tree was constructed using a matrix prepared with sequences of the 5′UTR region with sequences deposited in GenBank, including sequences from neighboring countries and the rest of the world, including the Paraguayan strains. Border Disease Virus and Pestivirus giraffe were used as outgroups. All sequences were aligned with ClustalW (v.7.1.3.0; BioEdit), and a phylogenetic tree was constructed using the Maximum-likelihood with Kimura+2+G model, as a statistician 1000 bootstraps were made in IQ-TREE and node supports are shown as SH-aLRT (%)/UFBootstrap (%), each based on 1000 replicates. The accession numbers of the sequences analyzed in this study were submitted to the GenBank database (PV987540 to PV987547).

## 3. Results

### 3.1. Bovine Semen and Serum Sample

**Figure 1:**
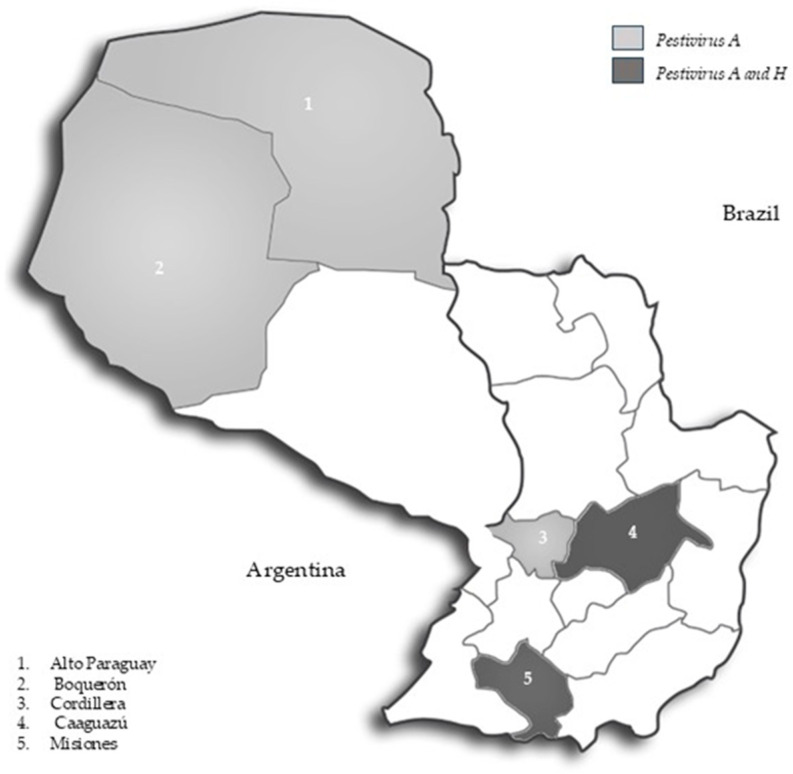
Geographical distribution of *Pestivirus A* and *H* in Paraguay.

Fifty five % (40/73) of the samples were positive for *Pestivirus A*, and all samples were negative for *Pestivirus B* and *H*. These samples were from the departments of Alto Paraguay, Boquerón, Caaguazú, and Cordillera and were collected from eight establishments for bovine reproduction purposes. It is important to note that the presence of *Pestivirus A* was detected in all the departments included in this study (Figure 1). ELISA results showed that 39.7% (29/73) of the animals were serologically positive and 60.3% (44/73) were negative (Table 1). Both techniques (semen RT-PCR and serum ELISA) were compared to determine whether there was any concordance between them. The Kappa index was −0.10, 95% CI (−0.32–0.11), between both techniques obtaining poor agreement, according to the analysis table of the Kappa index (<0.2 poor agreement).

### 3.2. Bovine Aborted Fetuses

Two of the eight fetuses studied were positive for *Pestivirus A* and Pestivirus H, and viral RNA was detected in the heart, liver, lung, and auricular cartilage of Fetus 1 and in the lung, auricular cartilage, brain, and liver of Fetus 7. Fetus 1 was from Caaguazú and was 4 to 6 months old (second trimester of gestation). Fetus 7 was from Misiones and was 7–9 months old (third trimester of gestation) (Table 2).

### 3.3. Sequencing and Bioinformatic Analyses

**Figure 2:**
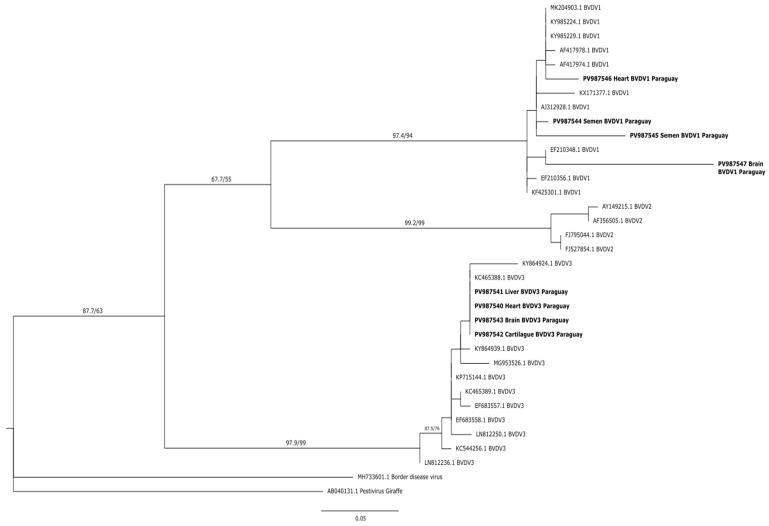
Phylogenetic tree based on the partial 5′UTR gene. Tree was generated using Maximum-likelihood with Kimura+2+G model, as a statistician 1000 bootstraps. Node supports are shown as SH-aLRT (%)/UFBootstrap (%) Paraguayan’s strain is marked in bold.

Phylogenetic reconstruction was performed using the maximum likelihood method, and the analysis clearly grouped the Paraguayan strains into two well-differentiated clusters, each corresponding to *Pestivirus* species *A* and *H*. Paraguayan sequences of the 5′UTR gene clustered with highly supported Pestivirus H strains (SH-aLRT 97.9%, UFBoot 99%). The Paraguayan strains corresponding to the H genotype formed a cluster similar to Brazilian strains. Strains of genotype *B* retrieved from Genbank were grouped into a single cluster. Strains corresponding to genotype A clustered in a clade with SH-aLRT 97.4% and UFBoot 94% support, and fetal samples (PV987546–PV987547) formed a separate cluster associated with strains from Argentina. Semen samples PV987544 and PV987545 formed a cluster associated with strains from Ireland and the UK. For a better analysis of the intragenotypic diversity of circulating BVDVs in Paraguay, it will be necessary to analyze a greater number of sequences and a variable region (such as the E gene) or perform whole genome sequencing (Figure 2).

## 4. Discussion

To the best of our knowledge, this is the first study to report the detection and characterization of viral RNA from BVDV in semen and fetal samples in Paraguay. In 73 semen samples analyzed, 54.7% (40/73) of animals were positive for *Pestivirus A*, resulting in negative genotypes *B* and *H*. BVDV can be transmitted by bulls, from natural mating or artificial insemination [2] and after an acute infection, the virus remain between 7 months and 2 years in the seminal material [22]. BVDV was detected in seminal samples in Brazil, India and Iran [23,24,25], nevertheless the prevalence of these studies was lower than the prevalence obtained in this study. This difference may be due to the fact that the establishments studied in Brazil had a sanitary plan for the control of the disease, and the animals were immunized [23]; however, in this study, we did not have epidemiological data of immunization and sanitary control of the establishments, which could indicate an important presence and viral dissemination in the herds studied.

Comparing RT-PCR and ELISA tests in semen and serum samples from bulls, poor agreement (Kappa < 0.2) was observed in relation to the presence of antibodies and RT-PCR. This may be due to the diversity of viral distribution, clinical presentation of the disease, and the immune status of males. BVDV can be eliminated in the semen of PI bulls, bulls in an acute infection, and also from animals with a persistent testicular infection [26] PI bulls eliminate high amounts of virus due to specific replication at the prostate and seminal vesicle level [27]. In animals with acute infection, the virus can be detected in semen after the viremic phase and when antibodies are detectable; thus, bulls develop a normal immune response with high titers of neutralizing antibodies. Therefore, all batches of semen from animals seropositive to BVDV must be tested with RT-PCR to exclude the possibility of being viral carriers [28]. Persistent testicular infection has been identified in non-viremic seropositive animals; thus, the virus is detected in semen but not in serum, with the presence of detectable antibodies, determining persistent testicular infection [26], This type of infection is usually characterized by a low prevalence and may be due to the specificity of the circulating strains, the routes and characteristics of the viral infection, the sexual maturity of the bulls at the time of infection, the immunocompetence of the host, and the stability of the blood-testicular barrier [29].

Two of eighth fetuses studied were positive for *Pestivirus A* and *Pestivirus H*, and viral RNA was detected in the heart, liver, lung, and auricular cartilage. These organs are generally the organs of choice for the diagnosis of abortion in ruminants; however, it is important to note that BVDV is frequently detected in fetuses that have miscarried for other reasons. This is because its immunosuppressive effect can exacerbate susceptibility to other pathogens [30]. Cases of abortion related to genotypes *A* (1a) and *B* (2b) have been identified in Uruguay, and it was determined that both genotypes have the same tissue tropism and that genotype *B* crosses the placenta faster than genotype *A* [31,32]. In this study, viral detection was performed in fetuses between the second and third trimesters of gestation, which is consistent with another study conducted in Argentina, in which a 64.9% viral detection rate was observed in aborted fetuses in the third trimester of gestation [33]. On some occasions, due to the difficulties in the referral of samples from aborted fetuses, the remission of fetal fluids (pleural or peritoneal fluids, plasma, or serum) is suggested [34]; however, in this study, no viral presence was detected in this type of sample studied, which may be due to the limited number of samples. Studies conducted by Oem et al. [35] detected 15.5% viral RNA in brain samples using RT-PCR, with genotypes A (1a and 1b) and B (2a) being the most prevalent. The H genotype has been detected in tissue samples (placenta, lung, spleen, liver, and kidney) from naturally aborted fetuses in Italy [36]. In contrast to these findings, this study reports, for the first time, the presence of this genotype in brain and heart samples, determining the wide tissue tropism of the virus. In this study, co-infection with two BVDV genotypes (*A* and *H*) was detected. Dual infections can occur naturally, and their distribution in tissues is different. The detection of both genotypes in fetuses provides evidence that infections occur naturally and are important as they are a source of viral recombination and can facilitate rapid genetic diversification, increasing virulence, or evasion of vaccine protection [37,38].

In this study, the co-circulation of two BVDV genotypes corresponding to three species of Pestivirus (*A* and *H*) was detected. The analyses of the 5′UTR region allow the correct location of the pestiviruses in species and, in some cases, define them in sub-genotypes. The disadvantages of their use for phylogenetic analyses are the restricted length of the sequence, the lack of diversity, and consequently, the lack of information that does not allow inferring the relationships between major clades, and some branches of the tree are poorly supported by statistical values [5]; however, in this study, the statistical values exceeded 90%. Phylogenetic studies of this region in this work showed that the 5′UTR correctly classified each corresponding genotype, although there is currently no target region considered standard for the classification of Pestivirus [39]. The organ sequences analyzed were associated with regional sequences from Argentina and Brazil; however, the semen sequences were associated with sequences from Ireland. This information allows us to determine that the circulation of the virus that especially affects cases of abortion that are circulating in the region of South America [5,13,32]. In future research, it is recommended to increase the number of fetal samples to more accurately determine which organs should be submitted for laboratory analysis for molecular diagnosis. Additionally, conducting paired sampling of specimens is suggested to ascertain persistent infections. It is also advised to perform sub-genotyping through the amplification of alternative target genes and to genotype vaccine strains to assess their coverage.

## 5. Conclusions

Two genotypes (*A* and *H*) of BVDV were detected for the first time in Paraguay using molecular techniques (RT-PCR) in semen and fetal organs (heart, liver, lung, brain, and auricular cartilage) with 5′UTR target gene. More research is needed to determine the epidemiology of this disease in our country. It is recommended to conduct comprehensive sampling across the entire national territory, ensuring a sample size that accurately represents the total cattle population. Additionally, it is imperative to investigate clinical cases that are consistent with the disease using the methodologies proposed in this study, specifically RT-PCR. Furthermore, it is crucial to inform the relevant authorities and producers about the presence of BVDV in Paraguay.

## Figures and Tables

**Table 1:** Results obtained by serology and RT-PCR in serum and semen samples from the bulls.

		Seminal Samples	Serum Samples
	*N*	*Pestivirus A* + RT-PCR	ELISA+
**Breed**			
Braford	5	5/5 (100%)	2/5 (40%)
Brahman	12	4/12 (33.3%)	0
Brangus	41	25/41 (61%)	21/41 (51%)
Pampa	2	Negative	2/2 (100%)
Senepol	4	3/4 (75%)	1/4 (25%)
Hibrid	9	3/9(33.3%)	3/9 (33%)
**Age**			
0–5 years	16	11/16 (69%)	5/16 (31%)
6–9 years	23	8/23 (35%)	11/23 (48%)
Adults	33	21/33 (64%)	13/33 (39%)
NDA *	1	Negative	0
**Department**			
Alto Paraguay	10	10/10 (100%)	4/10 (40%)
Boquerón	33	8/33 (24.2%)	15/33 (45%)
Caaguazú	10	9/10 (90%)	2/10 (20%)
Cordillera	20	13/20 (65%)	8/20 (40%)

* NDA: No data available.

**Table 2:** Fetal organs analyzed by RT-PCR, for the detection and genotyping of BVDV.

Organs	Fetus 1			Fetus 7		
	*Pestivirus*			*Pestivirus*		
	*A*	*B*	*H*	*A*	*B*	*H*
Heart	+	−	+	−	−	−
Lung	+	−	+	+	−	−
Liver	+	−	+	−	−	+
Brain	*	*	*	+	−	+
Auricular cartilage	+	−	+	−	−	+
Abdominal cavity liquid	−	−	−	*	*	*
Pericardiac Liquid	−	−	−	*	*	*

(+): Positive, (−): Negative, (*): Samples not processed.

## Data Availability

The original contributions presented in this study are included in the article. Further inquiries can be directed to the corresponding author.
