# Correction: Rodriguez Valinotti et al. Molecular Detection and Characterization of Bovine Diarrhea Virus (BVDV) in Aborted Fetuses and Semen Samples from Paraguay. *Viruses* 2025, *17*, 1295

**DOI:** 10.3390/v17111490

**Published:** 2025-11-11

**Authors:** María Fátima Rodriguez Valinotti, Eva Megumi Nara Pereira, Magaly Martinez Pereira, Rosmary Rodriguez Valinotti, Antonio Rodriguez Sanchez

**Affiliations:** 1 CEDIVEP, Veterinary Diagnostic Center of Paraguay, San Lorenzo 111422, Paraguay; r.rodriguez@cedivep.com.py (R.R.V.); arodriguez@cedivep.com.py (A.R.S.); 2 Molecular Biology and Biotechnology Department, Instituto de Investigaciones en Ciencias de la Salud, Universidad Nacional de Asunción, San Lorenzo 111422, Paraguay; enara@iics.una.py (E.M.N.P.); magaly.martinez@gmail.com (M.M.P.)

In the original publication [1], the Institutional Review Board Statement was incorrect. The Informed Consent Statement and the Data Availability Statement were not included. The authors stated that all experiments in the study were conducted in full compliance with the relevant ethical standards, and the corresponding ethics approvals had been obtained prior to the study. The corrected statements are provided below.

**Institutional Review Board Statement:** The full protocol of this research has been approved by the Research Ethics Committee and the Scientific Committee of the Instituto de Investigaciones en Ciencias de la Salud (Approval code: P05/2019, approval date: 16 July 2019).
**Informed Consent Statement:** Not applicable.
**Data Availability Statement:** The original contributions presented in this study are included in the article. Further inquiries can be directed to the corresponding author.

The authors state that the scientific conclusions are unaffected. This correction was approved by the Academic Editor. The original publication has also been updated.

## References

[1] Rodriguez Valinotti M.F., Nara Pereira E.M., Martinez Pereira M., Rodriguez Valinotti R., Rodriguez Sanchez A. (2025). Molecular Detection and Characterization of Bovine Diarrhea Virus (BVDV) in Aborted Fetuses and Semen Samples from Paraguay. Viruses.

